# Interfacial Layer Breaker: A Violation of Stokes’ Law in High-Speed Atomic Force Microscope Flows

**DOI:** 10.1021/acs.langmuir.2c02418

**Published:** 2022-12-20

**Authors:** Fan Li, Stoyan K. Smoukov, Ivan Korotkin, Makoto Taiji, Sergey Karabasov

**Affiliations:** † The School of Engineering and Materials Science, Queen Mary University of London, Mile End Road, E1 4NSLondon, United Kingdom; ‡ Mathematical Sciences, University of Southampton, University Road, SO17 1BJSouthampton, United Kingdom; § Laboratory for Computational Molecular Design, Computational Biology Research Core, RIKEN Quantitative Biology Center (QBiC), 1-6-5 Minatojima Minamimachi, Chuo-Ku, Kobe, Hyogo650-0047, Japan

## Abstract

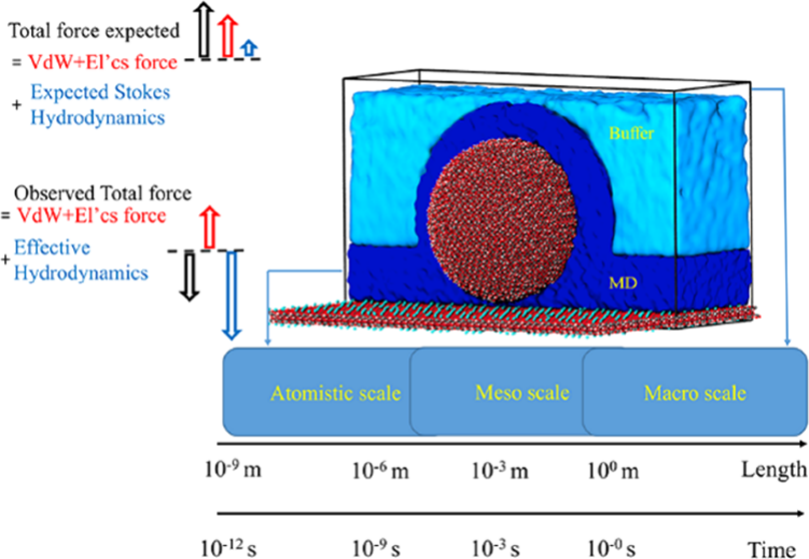

Structured water near surfaces is important in nonclassical crystallization, biomineralization, and restructuring of cellular membranes. In addition to equilibrium structures, studied by atomic force microscopy (AFM), high-speed AFM (H-S AFM) can now detect piconewton forces in microseconds. With increasing speeds and decreasing tip diameters, there is a danger that continuum water models will not hold, and molecular dynamic (MD) simulations would be needed for accurate predictions. MD simulations, however, can only evolve over tens of nanoseconds due to memory and computational efficiency/speed limitations, so new methods are needed to bridge the gap. Here, we report a hybrid, multiscale simulation method, which can bridge the size and time scale gaps to existing experiments. Structured water is studied between a moving silica AFM colloidal tip and a cleaved mica surface. The computational domain includes 1,472,766 atoms. To mimic the effect of long-range hydrodynamic forces occurring in water, when moving the AFM tip at speeds from 5 × 10^–7^ to 30 m/s, a hybrid multiscale method with local atomistic resolution is used, which serves as an effective open-domain boundary condition. The multiscale simulation is thus equivalent to using a macroscopically large computational domain with equilibrium boundary conditions. Quantification of the drag force shows the breaking of continuum behavior. Nonmonotonic dependence on both the tip speed and distance from the surface implies breaking of the hydration layer around the moving tip at time scales smaller than water cluster formation and strong water compressibility effects at the highest speeds.

## Introduction

The intricacy of water structure at nanoscale distances from a material surface has been observed by both experiments and equilibrium molecular dynamic (MD) simulations.^1−6^ The study of this structure is important in nonclassical crystallization,^7−9^ biomineralization,^10^ and tribochemical wear.^11^ Interfacial water properties are also pertinent for the restructuring processes of cellular membranes^12−14^ and organismal signaling.^15^ The advent of advanced high-speed atomic force microscopy (H-S AFM) opens new opportunities for measuring the dynamic piconewton forces, which occur within microseconds and nanometer structural rearrangements with subsecond resolution.^16,17^ AFM tools are used to detect the equilibrium structure of water layers, but the dynamic behavior is still beyond the experimental reach in the submicrosecond and nanosecond regions typical of structural rearrangements and biocatalytic molecular systems.^18−20^ While the time step of molecular dynamic (MD) simulations is orders of magnitude too small and so are their system sizes to model such processes, which involve collective dynamics of many water molecules, the hybrid multiscale methods, which combine MD with continuum fluid dynamic simulations,^21−24^ provide an attractive opportunity for the modeling of realistic-size systems while resolving details of the molecular ordering. Here, we report the detection of water structuring between a silica AFM colloidal tip and a cleaved mica surface. The quantification of the drag force on the tip as a function of the tip–wall distance and tip velocity shows nonmonotonic force, which becomes negative for high-speed regimes, implying variations in not only viscosity but also density. At the highest speeds, these are consistent with the breaking of the hydration layer around the moving tip and formation of a layered water structure in the subnanometer gap between the tip and the substrate surface.

## Methods

**Figure 1:**
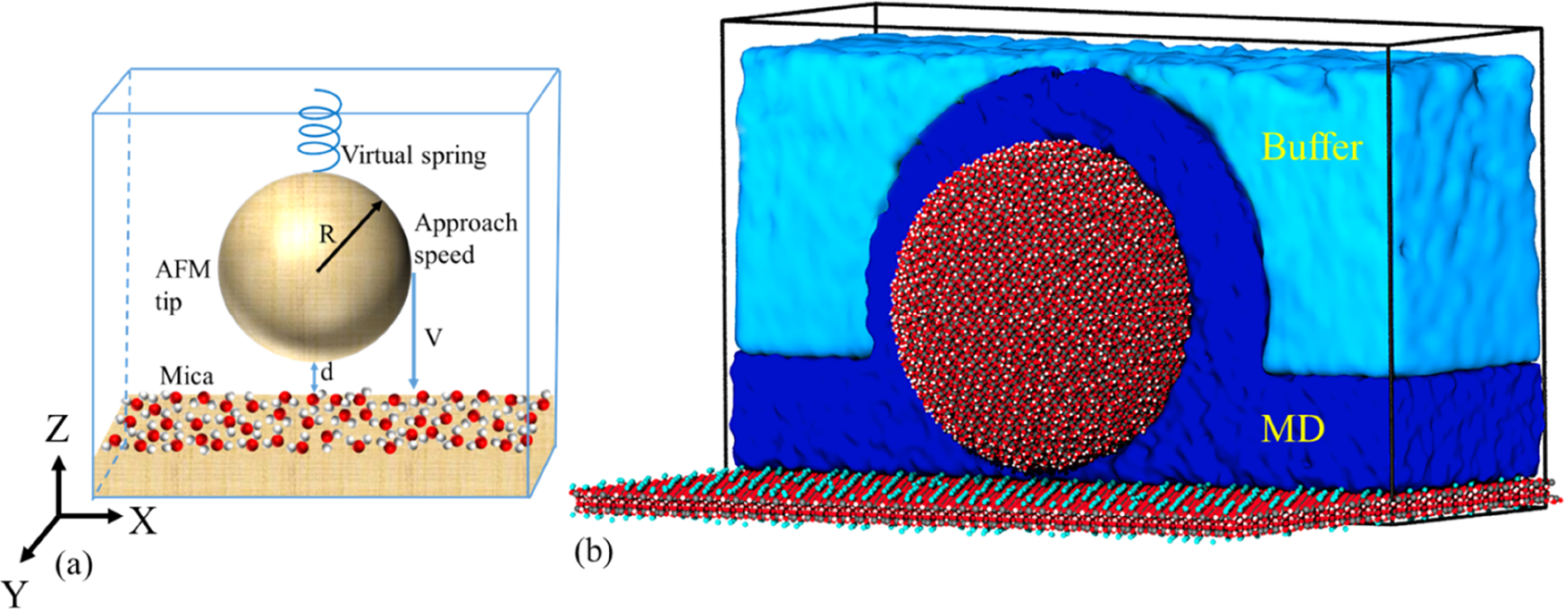
(a) Schematic representation of one-half of the symmetric computational domain (26 nm x 19 nm x 15.6 nm). The symmetry plane corresponds to the top boundary in the *z*-direction. The silica tip is represented by a sphere (*R* = 5 nm), and *d* is a distance between the tangential plane of the sphere and the potassium ion (K) layer of the mica surface. The sphere is suspended by a massless spring from the top of the box, and the resulting force *F* on the tip is measured in accordance with Hooke’s law, *F* = −*k*·Δ*z*, where *k* and Δ*z* are the virtual spring constant and the displacement, respectively. The approach speed *V* of the tip is varied. (b) A cut through the simulation domain showing the decomposition of the hybrid multiscale simulation domain into several regions. The silica tip and mica surface are modeled at the full-atom resolution. In addition, a part of the water volume in a rectangular slab, which covers the mica surface with a 3.7 nm thickness, and the water in a spherical shell, which covers the silica tip with a 2.79 nm thickness, are also modeled at the all-atom resolution (water in the pure atomistic MD region is shown in dark blue). Outside of the pure atomistic region, a multiscale interface region is placed where the MD particles are subjected to a hydrodynamic force including the Brownian motion. The thickness of the multiscale region is selected to include at least a few cutoff radii of van der Waals forces for best performance (see the Supporting Information for further details). The multiscale interface and the outer region, where water molecules are fully dominated by hydrodynamic forces (buffer), are shown in light blue. The hydrodynamic forces are consistent with the macroscopic Stokes solution in a large water volume surrounding the MD domain so that no reflection of pressure waves occurs at the boundaries of the multiscale domain.

Figure 1a shows the schematic representation of the simulated AFM device, where a silica tip functionalized by the hydroxy group on the surface with a radius of 5 nm that corresponds to a mid-range tip size compared to the values reported in the AFM experiments.^25,26^ The tip is attached by a massless virtual spring and suspended above the mica substrate at a distance *d*. The simplification of the AFM device as a massless spring attached to a spherical tip is a well-established practice in molecular modeling for it simplifies the comparison of the numerical simulation with the analytical Stokes’ solution in the continuum hydrodynamic theory.^16,27^ In both cases, the drag force acting on a sphere moving in viscous liquid is considered. Details of the force comparison between the results of the multiscale calculations and predictions based on Stokes’ law are presented in the following paragraph. Following (28, 29), a detailed atomistic model of the mica substrate (K[Si_3_AlO_8_]Al_2_O_2_(OH)_2_) has been implemented. In the model, the Si → Al charge defects are distributed in agreement with the solid-state nuclear magnetic resonance data.^30^ The silica tip and mica substrate are fully immersed in water. For water–mica, water–tip, and silica–mica modeling, the recently developed INTERFACE force field is applied, which was shown to provide good results for interfacial systems.^31^Figure 1b illustrates the partition of the simulation domain in the multiscale model, where the external boundary conditions in the continuum part of the simulation are prescribed in accordance with the macroscopic Stokes model by introducing the fluctuating hydrodynamic (FH) force in the equations of MD particles. The center of the domain including the water layers on material surfaces is simulated at the all-atom resolution; hence, all of the interactions at the all-atom level are accurately captured as in the conventional MD method. The continuum flow effects are enforced by the moving tip via the effective boundary conditions using the multiscale method (see the method details in the Supporting Information). Therefore, the input parameters of the model are the tip–substrate distance, *d*, and the AFM speed, *V*, while the output is the force on the AFM tip (Figure 1).

It should be noted that the bulk hydrodynamic flow effect induced by the moving AFM boundary decays slowly with the distance. Furthermore, the simulation time needed for averaging out the thermal velocity fluctuations of water atoms (∼100 m/s), which amplitude is much larger than the hydrodynamic signal, is macroscopically large too. Altogether, this means that using the standard all-atom MD approach with equilibrium boundary conditions would require a very large computational domain in space and time, which is prohibitively expensive. Therefore, the implemented multiscale method essentially truncates the computational domain while preserving the atomistic resolution where it is critical to account for short-range interatomic interactions.

To calculate the total force exerted on the AFM tip, individual forces in the all-atom region from the surrounding water atoms and the mica substrate are summed up and time-averaged. The averaging volume comprising the water and mica atoms around the AFM tip, which defines the force exerted on the AFM tip, is governed by short-range interatomic interactions between those atoms and the microscopic AFM tip. This microscopic volume is the effective volume, which corresponds to the statistical ensemble averaging used in the current simulations. The collective force associated with the pure hydrodynamic effect is computed as a difference between the total AFM force with and without the AFM tip motion.

To validate the FH/MD model, the tip velocity is set to zero, and the resulting force–distance curve solution is compared with the solution obtained from the all-atom molecular dynamic simulation performed for the same system with periodic boundary conditions. The force is calculated in accordance with Hooke’s law, *F* = −*k*·Δ*z*, where *k* and Δ*z* are the virtual spring constant and the displacement in the *z*-direction, respectively. The distance *d* in the force–distance curve is defined as the distance between the tangential plane of the sphere and the potassium ion (K) layer of the mica surface. Notably, as shown in Figure S1a in the Supporting Information, the difference between the FH/MD and the reference all-atom MD solution is within 0.05 nN. Furthermore, the sensitivity of the MD solution to the averaging window, 2 vs 3 ns, is also approximately within the same 0.05 nN error bar (see Figure S1b). It can be noted that this difference could be further reduced by running the simulations for a longer time to better converge the models statistically.

The above error of the multiscale model solution is much smaller than the uncertainty of the computed force associated with the thermal fluctuations of water molecules in the volume corresponding to the tip and the typical averaging time of MD solutions. On the one hand, the uncertainty in the AFM force measurement due to the Brownian motion can be estimated as the difference between the forces applied on the AFM sphere and its mirror image in the upper part of the symmetrical computational domain (which fluctuates randomly for different AFM distances and speeds in accordance with the random nature of the Brownian force, as shown in Figure S2 in the Supporting Information). On the other hand, for the case when the AFM tip is approaching the mica surface slowly and is considered at a distance greater than the hydration layer from the mica surface, the uncertainty in the force associated with the Brownian motion can be estimated as a perturbation of Stokes’ law, δ*F* ∼ 6π*R*μ·Var(*V*′). Here, μ is the dynamic viscosity of bulk water and *V*′ is the thermal velocity fluctuation. The variance corresponds to averaging over the grand-canonical ensemble in accordance with the fluctuating hydrodynamic theory,^21^, where *V*_0_ = 4π*R*^3^/3 is the characteristic control volume, *k*_B_ is the Boltzmann constant, *T* is the temperature, and ρ_0_ is the bulk water density at equilibrium conditions. For a large AFM distance of 0.77 nm and a low speed of 5 × 10^–7^ m/s, where Stokes’ law should be approximately valid, the theoretical estimate gives δ*F* = 0.234 nN. It can also be noted that the latter uncertainty associated with the Brownian motion in the small molecular system considered is in the order of the positive drag force predicted by the continuum Stokes’ theory at medium–high AFM speeds (*F*^Stokes^ = 0.283 nN at 0.3 m/s). This explains why the classical positive hydrodynamic drag effect is not resolved in the AFM force–distance curve (Figure S2).

Furthermore, the estimated uncertainty due to the Brownian motion is in good agreement with the numerical simulation result, i.e., one-half of the magnitude of the force difference between the AFM sphere and its mirror image in the same conditions, 0.3 nN (Figure S2 in the Supporting Information). Hence, in the remaining part of the article, the numerically computed one-half of the absolute difference between the computed forces on the AFM tip and its mirror image will be used as an estimate of the standard deviation of the statistical error for each tip speed and mica–tip distance.

Results of additional calculations with the FH/MD method for a test molecular liquid system are included in the Supporting Information (Figure S5). The test system shares a few pertinent features with the water flow over the AFM tip, such as flows through the hybrid FH/MD interface region, while allowing a direct comparison with a reference all-atom MD solution in this case. The comparison shows that the moving multiscale interface does not lead to noticeable numerical artifacts in computing the molecular diffusion coefficient.

## Results

**Figure 2:**
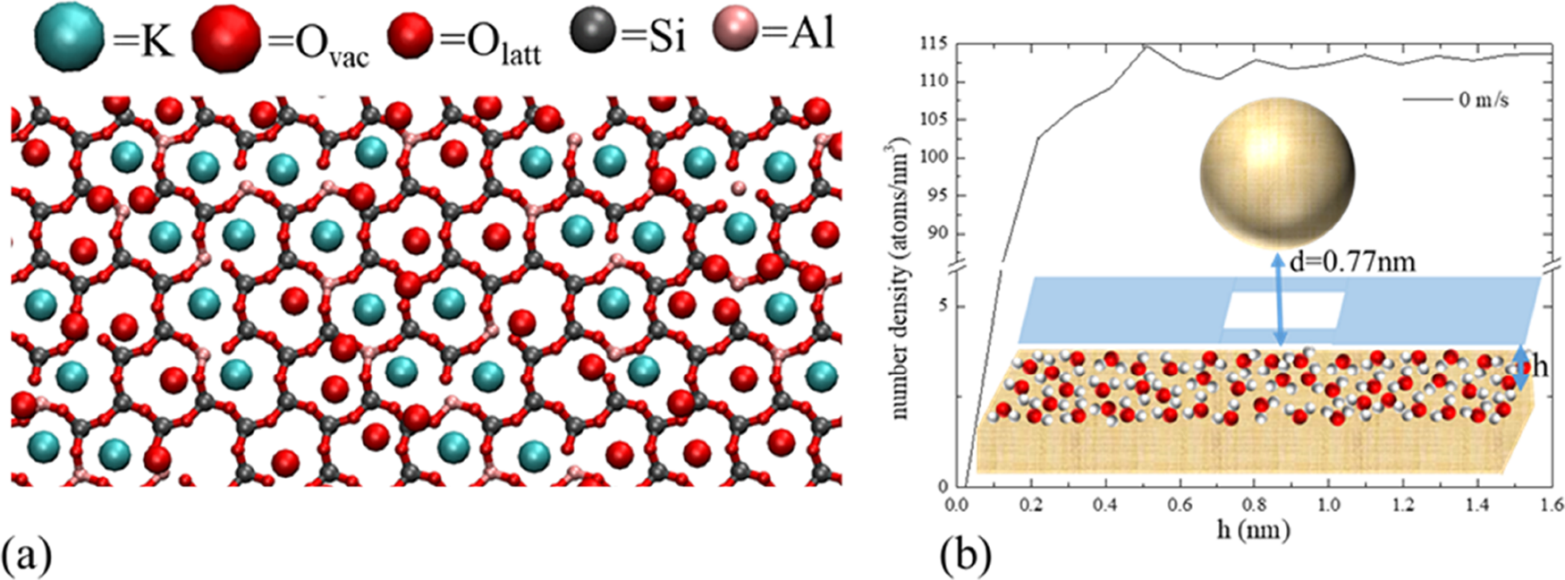
(a) First mica layer in contact with the water. The interface is composed of potassium ions from the mica (blue) and oxygen atoms adsorbed in vacant potassium cavities in the mica (bigger red *O*_vac_). Oxygen (smaller red *O*_latt_), silicon (gray), and aluminum (pink) make the rest of the mica lattice. (b) Plot of the average number density of water atoms in planar 0.05 nm thick slices immediately above the mica (and excluding the 10 nm × 10 nm square area below the sphere, (white)) as a function of the slice’ height, *h*, in the *z*-coordinate direction. The potassium ions’ plane of the mica surface is taken as *h* = 0, and densities are plotted up to a height of 1.6 nm. The mica–tip distance from the same reference is *d* = 0.77 nm.

The properties of water close to the mica surface simulated with the FH/MD solution are analyzed separately in Figure 2. To reduce the effect of the AFM tip, the stationary regime is considered where the tip is fixed at a distance from the mica of *d* = 0.77 nm, which is much larger than the radius of van der Waals forces. From Figure 2a, it can be noted that the water atoms are absorbed next to the mica cations, one per vacancy of the mica crystal, in accordance with the experimental and previous numerical studies.^32,33^ Notably, the periodic structure formed by the absorption of water atoms in the mica structure is crucial for the hydration layer formation. For example, as demonstrated in the atomic force microscope experiments using frequency modulation^2^ and equilibrium all-atom MD simulations,^4,34^ the hydration layer is responsible for the amplitude of the repulsive peak of the AFM force–distance curve at *d* = 0.5–0.6 nm when the tip velocity is zero (Figures 4b and S1a). Further discussion of the force–distance curve can be found in the Supporting Information.

Figure 2b shows the distribution of the number density of water in the (wall-normal) *z*-direction from the mica surface. The density profile is calculated by averaging the mass of water molecules contained in each vertical bin between the mica surface and the maximum height (0.05 nm) of the water column considered in the mica–tip gap over 2 ns. It should be reminded that, as in Figure 2a, the point of interest here is the water density profile close to the mica and away from the AFM tip. Therefore, to exclude the effects of nonuniform water confinement between the mica surface and the AFM tip, the water volume corresponding to the square area of 10 nm × 10 nm under the AFM tip was excluded from the averaging when computing the water density profile on mica. Notably, the resulting density profile has a prominent peak in the vicinity of the mica surface followed by several smaller peaks and deeps. Again, this is in good qualitative agreement with the typical interfacial water structure observed experimentally.

Having validated the force–distance curve of the multiscale model for the stationary AFM regime and shown the known water layering near a mica surface, we investigated the fate of water layering in a dynamic context. We performed a series of simulations for a range of approach speeds of the AFM tips from 5 × 10^–7^ m/s (typical speed of the AFM cantilever in experiments) to 30 m/s (hypothetical high-speed AFM regime).

**Figure 3:**
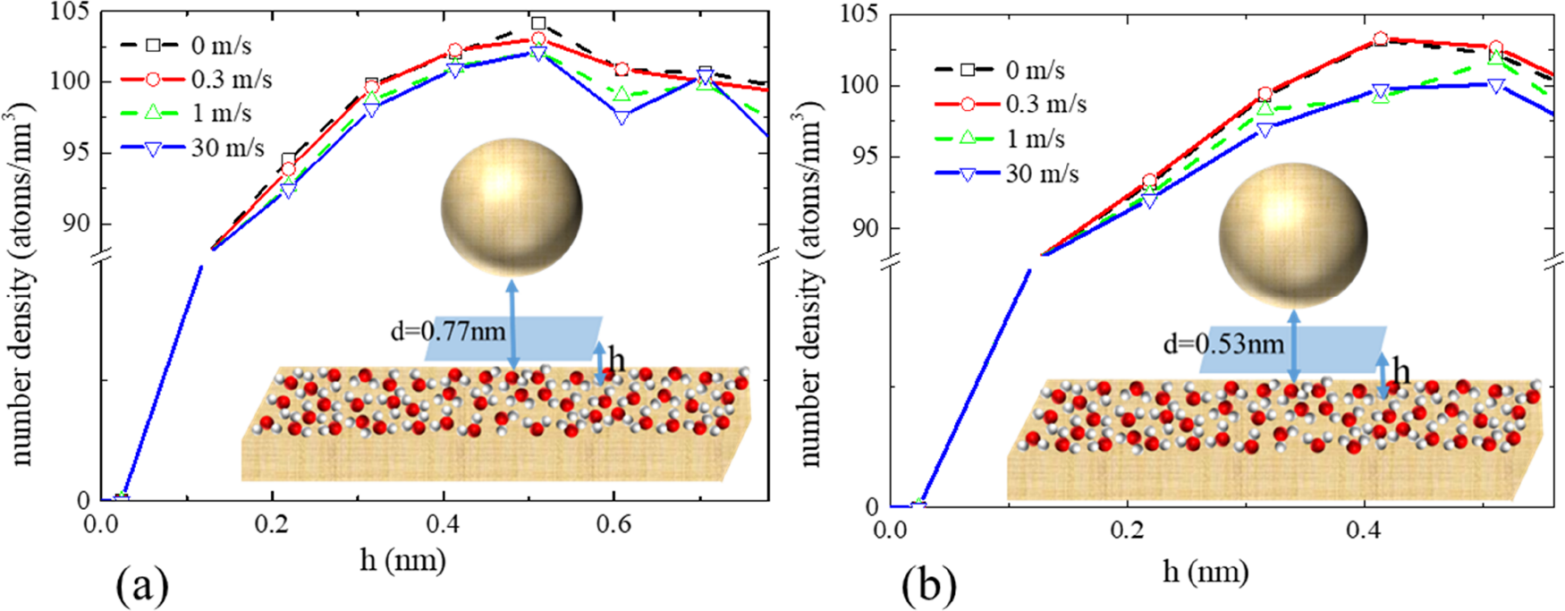
Averaged number density of water atoms in a planar slice below the tip sphere (blue, 10 nm × 10 nm) as a function of the slice’ height, *h*, in the *z*-coordinate direction; the blue area in this figure corresponds to the white area in Figure 2b. The bin thickness used in the averaging is 0.05 nm. Panels (a, b) correspond to 0.77 and 0.53 nm mica–tip distance, *d*, respectively. Note the distinctly layered structure of the water density distribution at a 30 m/s tip speed in panel (a).

We find that the layering under the tip close to the mica is disturbed due to the presence of the moving curved geometry. For the relatively large mica–tip distance, *d* = 0.77 nm, the higher the tip speed, the more layering could be observed, which is especially notable at 30 m/s, which corresponds to an oscillation in the density profile at around 0.6 nm in Figure 3a. Figure 3b shows the variation of water density with height for a different AFM tip–mica separation distance *d* = 0.53 nm and a range of approach speeds, 0–30 m/s. For such small mica–tip distance, regardless of the tip speed, the hydration layer cannot fit in the gap due to the proximity of the AFM tip.

In turn, the dynamic layering of water near the tip leads to significant, nonintuitive departures from the expected forces on the AFM tip in accordance with the continuum hydrodynamics.

**Figure 4:**
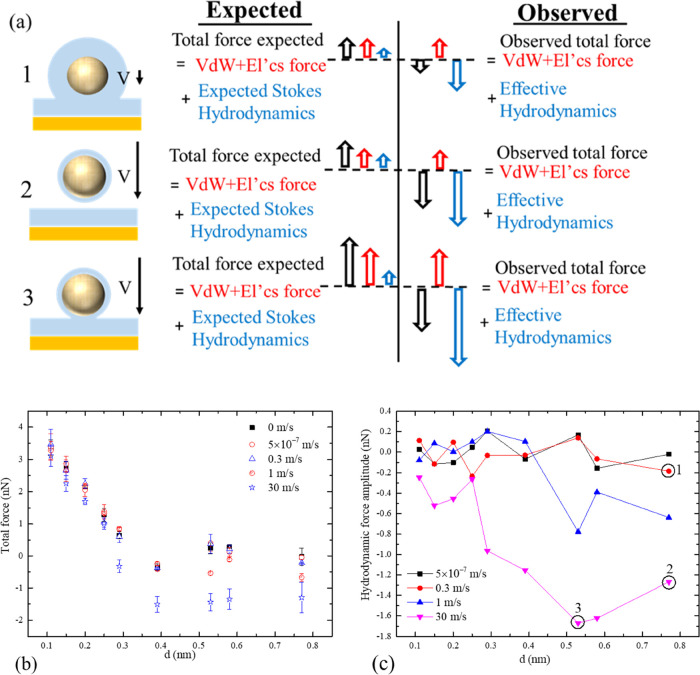
(a) Schematic of the sphere approaching the structured water layer above the mica plane; the thickness of the interfacial water layers depends on the mica–tip gap and the tip speed and comparison of the total tip force predicted by classical hydrodynamic theory and FH/MD. For *d* = 0.77 nm, at small speeds (denoted by Num. 1), a hydration layer occurs on the tip surface. At high speed (denoted by Num. 2), the hydration layer on the AFM tip breaks down because of water molecules depleting from it to bulk, thereby resulting in the occurrence of a layered water structure, which corresponds to the single hydration layer on the mica surface, while the hydration layer on the mica stays the same. As the tip approaches the mica at high speed even closer (denoted by Num. 3), both the hydration layer on the tip and the mica are broken down because of the confinement. Center and right: comparison of the total force based on the predicted hydrodynamic force from Stokes’ law and the numerical simulation result. (b) Total force on the AFM tip as a function of distance from the mica surface for velocities 0, 5 × 10^–7^, 1, and 30 m/s. The error bar is the absolute difference of the total force between the AFM tip and its symmetrical image on the top. The total force includes van der Waals (vdW) and electrostatic (El’cs) force from water and mica substrate for the zero tip velocity. For other velocities, the total force also includes the hydrodynamic force because of the incorporated flow. (c) The effective hydrodynamic drag force on the sphere, i.e., the difference between the computed AFM forces in the stationary regime and the force in the flow regime measured for the same mica–tip distance.

Simulation results for various tip/mica distances, *h*, and AFM speeds, *V*, are summarized in Figure 4b,c. Figure 4a provides a roadmap to these results in the form of sketches. On the left, three typical tip/mica configuration regimes are presented: (1) the equilibrium situation when the tip is moving at a low speed with hydration layers formed on both the surfaces and (2) and (3) when the tip moving at a high speed further or closer to the mica, so that the hydration layer on the tip does not have time to form. The second and the third columns show schematics of the force balances, where the total force includes both the conventional interatomic, van der Waals, and electrostatic forces, which mostly depend on the tip–mica distance and the collective effect of moving water molecules (the hydrodynamic force). The second column corresponds to the classical Stokesian hydrodynamics, which ignore the noncontinuum effects such as the hydration layer, thereby always predicting a positive total repulsion force on the tip. In accordance with this model, the hydrodynamic drag of a small sphere moving in a viscous liquid only depends on the liquid viscosity, sphere diameter, and speed but critically is in the direction opposite to the body velocity.^35^ The third column shows the actual balance of the forces in accordance with the simulation FH/MD results. In accordance with the multiscale simulations, the hydrodynamic force is not only of a different magnitude but also in the opposite direction compared to the Stokes theory. The potential role of slip was excluded as a cause, as according to the continuum theory, the drag force reduces by 1.5 times when the hydrophilic nonslip condition on the spherical tip surface is replaced by the hydrophobic full-slip.^36^ And regardless of the wall boundary condition on a moving sphere down, Stokes’ drag should always correspond to a repulsive force. However, the actual simulation results show an attractive hydrodynamic force. It can be hypothesized that the collective attractive force may be associated with destroying the hydration layers around the fast-moving microscopic body, which redistributes pressure around the tip by creating an upstream rarefaction zone in the water volume by analogy with the ice breaker.

The total forces experienced on the nanometer tip when moving at different speeds up to 30 m/s are shown with their error bars in Figure 4b. This data set is used to compute the hydrodynamic interactions by subtracting the well-defined interatomic (van der Waals and electrostatic) interactions from the total force. We plot the resulting hydrodynamic forces in Figure 4c, showing that they are nonmonotonic, large in magnitude, especially in the highest velocity case, 30 m/s, and opposite to their usual direction in accordance with the Stokes model. Typical flow regimes (1)–(3) discussed in Figure 4a are clearly marked on the force distribution curves.

The answer to the puzzling behavior of the hydrodynamic force lies in the violation of the continuum theory due to the breaking of the hydration layer on the moving tip surface. When the tip–mica distance *d* is in the range of a small integer number of hydration layers, the forces can no longer be described by the continuum theory. Further, the formation of such hydration layer structures is associated with the collective behavior of water clusters, which require a small but finite time to form, in the order of 10^–11^ s according to.^37^ At the same time, the tip approaching the mica surface at a speed of 30 m/s passes through the characteristic length scale of water structure, such as the equilibrium intermolecular distance between the oxygen atoms, 0.31 nm in about 10^–11^ s. This shows that, for the very high approach speeds, there is little time for the hydration layers to form on the AFM tip. This observation is consistent with the decreased number density around the tip, as shown in Figure S3 in the Supporting Information. Furthermore, there is another important physical time scale for this problem, which is related to water compressibility. As the water squeezed between the mica surface and the approaching tip is pushed and displaced to the tip sides, the propagating pressure waves need to cover the distance equal at least to the tip diameter (10 nm). Assuming the pressure waves propagate in water at a normal speed of sound conditions (1500 m/s), the time for a single wave to traverse the tip is also similar (0.67 × 10^–11^ s). This means the water compressed under the approaching AFM tip at a speed of 30 m/s does not have time to settle to the equilibrium pressure state, which would require many passing-through cycles of the pressure wave. Altogether, this suggests that, depending on the tip speed and size, the water in the subnanometer gap between the approaching tip and the mica can be highly compressible, thereby potentially leading to the formation of strong nondivergent flows and rarefaction under the tip. In turn, this generates a negative pressure zone below the spherical tip, which cannot be explained by the classical Stokes model that assumes water incompressibility. The compressibility of water is another potential cause for the oscillation in the water density at a 30 m/s tip speed, as shown earlier in Figure 3a. In accordance with the numerical solution, the characteristic size of this oscillation, which occurs close to the AFM tip surface, is about 0.2 nm. By combining this space length with the tip speed (30 m/s), one obtains the corresponding time scale of the oscillation, 0.67 × 10^–11^ s, which is remarkably close to the water compressibility time scale discussed above.

## Conclusions

In conclusion, we demonstrate a hybrid continuum—molecular dynamic (FH/MD) method—capable of simulating 1,472,766 atoms, in a 7700 nm^3^ box size system for 2–3 ns with MD-like precision, while the outer continuum part of the model, which drives the continuum bulk-flow effects, is many orders of magnitude larger. Hence, the key feature of the multiscale solution used here is that it allows bridging the gap in time scales between the fast atoms/thermal fluctuations and the slow continuum/AFM motion.

Notably, the cantilever structure above the AFM tip in the multiscale simulation was approximated by a massless spring model consistently with the molecular dynamic literature.^16,27^ This is a reasonable approximation because it is equivalent to focusing on the localized flow effects around the microscopic tip, which exhibit the non-Stokesian behavior due to the breach of the continuity assumption at high-speed AFM regimes. These effects are treated as a perturbation to the continuum bulk flow imposed away from the moving AFM tip. At the same time, the size of the full AFM system does not influence the simulation time significantly, as any increases beyond the immediate vicinity of the mica and the tip are treated only by continuum mechanics. This is because (1) majority of the atomistic forces are short-ranged (a few nanometers) and (2) the important length scales of the problem such as the AFM tip radius and the distance between the AFM tip and the substrate wall are 5 nanometer and a subnanometer, respectively. Therefore, in static conditions, the entire AFM system could be modeled at the all-atom resolution within a modest MD box at equilibrium boundary conditions. What cannot be modeled by pure MD methods is the slow motion of the AFM tip, which generates a substantial bulk-flow hydrodynamic effect on the boundary conditions of the water atoms surrounding the AFM tip. Because this hydrodynamic effect is much slower than thermal fluctuations, one would require prohibitively long-time averaging/MD simulation times to separate this collective effect from thermal noise. At the same time, the simulation of the bulk-flow effects using a continuum fluid dynamic method is many orders of magnitude faster than the equivalent all-atom MD model and speed-up is utilized in the suggested multiscale model. For a 5 nm sphere, 0.5–0.77 nm from a mica surface, moving toward it at 1–30 m/s, we show that the continuum Stokes’ law expression for drag force fails spectacularly. Rather than a drag force opposite the direction of motion, the effective hydrodynamic force is in the direction of the motion and can be significantly larger than the respective electrostatic and van der Waals contributions. The collective force of water molecules deviates from the continuum, where the time to rearrange molecules during the high-speed AFM tip motion falls below the time of formation of water clusters and the time for the water density around the tip to equilibrate. With a decrease in tip sizes and higher frequencies, current high-speed AFM (H-S AFM) experiments are starting to approach velocities considered here. FH/MD simulations would be critical for interpreting the force components once the operation velocity in future advanced H-S AFM experiments reaches 1 m/s.

The current simulation results can be viewed as a high-speed extension of recent experimental observations from (38), which revealed that the atomic-scale features observed in force–distance curves in AFM experiments reflect the inhomogeneous perturbation introduced by the tip and the substrate in the liquid.

## Supporting Information Available

FH/MD method and its validation for the force–distance AFM curve; and validation of the FH/MD method for a test molecular system with moving the atomistic/continuum interface (PDF)
