# Supplementary material for: Interfacial Layer Breaker: A Violation of Stokes’ Law in High-Speed Atomic Force Microscope Flows
Source: Langmuir. 2022 Dec 20;39(1):220–6. doi: 10.1021/acs.langmuir.2c02418 (PMC9835886; doi:10.1021/acs.langmuir.2c02418)
Supplement: Supplementary file 1 — la2c02418_si_001.pdf [file la2c02418_si_001.pdf]

Supporting Information

The interfacial layer breaker: a violation of Stokes' law in high-speed Atomic Force Microscope flows

Fan Li[1], Stoyan K. Smoukov[1], Ivan Korotkin[1,2], Makoto Taiji[3], and Sergey Karabasov[1*]

[1] The School of Engineering and Materials Science, Queen Mary University of London, Mile End Road, E1 4NS London, United Kingdom

[2] Mathematical Sciences, University of Southampton, University Road, SO17 1BJ Southampton, United Kingdom

[3] Laboratory for Computational Molecular Design, Computational Biology Research Core, RIKEN Quantitative Biology Center (QBiC), 1-6-5 Minatojima Minamimachi, Chuo-Ku, Kobe, Hyogo, 650-0047 Japan

Corresponding author: s.karabasov@qmul.ac.uk

Contents

1. The method and its validation for the force-distance AFM curve
2. The fluctuations of the force-distance curve due to Brownian motion and the water number density above the moving AFM tip depending on the tip speed
3. Validation of the hybrid FH/MD method for a test molecular system with moving the atomistic/continuum interface

1. The method and its validation for the force-distance AFM curve

To simulate the effect of an oscillating cantilever, we impose the appropriate flow conditions in accordance with the continuum flow solution, which is smoothly enforced on the MD particles thanks to the use of the multiscale method. Instead of moving the AFM tip boundary in the simulation, for each approach velocity regime, the equivalent macroscopic flow near the AFM tip is generated by specifying the suitable boundary conditions in the continuum part of the simulation domain away from the tip. Such a method neglects both the nonlinear inertia and unsteady effects, in accordance with Stokes' flow, which is expected to hold away from the tip. This approximation is justified since even for the highest AFM speed considered (30 m/s), the Reynolds number of the bulk water flow induced by the AFM motion remains much smaller than unity due to the small AFM tip size ($R = 5\ nm$). At the same time, this effective boundary condition treatment helps avoiding the 'curse' of the very slow statistical convergence of Non-Equilibrium MD simulations, which would make one to enforce unrealistically high flow velocities (on the order of the thermal velocity fluctuations) to correctly capture the bulk water flow effects [1].

The continuum hydrodynamic flow is solved for as a combination of the steady incompressible flow and the unsteady compressible component, which occurs due to the thermal fluctuations of water. The fluctuations are important for the nanometer-size control volume including the interfacial water layers on all material interfaces. The

latter layers are modelled at the all-atom resolution. The incompressible flow solution is obtained by analytically solving the Stokes equation for the sphere moving normally to the substrate wall using the bipolar coordinate transformation method [2]. The fluctuating part of the continuum flow solution is obtained by numerically solving the Landau-Lifshitz Fluctuating Hydrodynamics (LL-FH) equations using the finite-volume method [3] implemented in GROMACS. Because of the scale separation between the Stokes flow and the thermal fluctuations, the entire solution is represented by

$$(\rho,\mathbf{u})^{total}(\boldsymbol{x},t) = \left(\rho(\boldsymbol{x},\mathrm{t}),\mathbf{u}^{Stokes}(\boldsymbol{x}) + \mathbf{u}'^{(\boldsymbol{x},\mathrm{t})}\right)\#(1)$$

where $\rho(\boldsymbol{x},\mathrm{t}) = \rho_0 + \rho'(\boldsymbol{x},\mathrm{t})$, $\left(\rho_0,\mathbf{u}^{Stokes}(\boldsymbol{x})\right)$ is the Stokes solution for the sphere/wall problem and $\left(\rho'(\boldsymbol{x},\mathrm{t}),\mathbf{u}'(\boldsymbol{x},\mathrm{t})\right)$ corresponds to the thermal density and velocity fluctuations about the uniform background $\left(\rho_0,\mathbf{u}_0 = 0\right)$.

The density and fluctuating velocity $(\rho(\boldsymbol{x},\mathrm{t}),\mathbf{u}'(\boldsymbol{x},\mathrm{t}))$ satisfy the LL-FH equations,

$$\frac{\partial \rho}{\partial t} + \mathrm{div}(\rho \cdot \mathbf{u}') = 0\#(2)$$

$$\frac{\partial\left(\rho \cdot u_i'\right)}{\partial t} + \mathrm{div}\left(\rho \cdot u_i' \cdot \mathbf{u}'\right) = \sum_{i,j\,=\,1,3} \nabla_j\left(\Pi_{ij} + \Pi'_{ij}\right)\#(3)$$

Here the Reynolds stress and its fluctuation component, Π_{ij} and Π'_{ij} are given by

$$\Pi_{ij} = -\,p'(\rho)\delta_{ij},\#(4)$$

$$\Pi'_{ij} = \xi \mathrm{div}\ \mathbf{u}'\delta_{ij} + \eta\left(\partial_i u_j' + \partial_j u_i' - 2D^{-1}\mathrm{div}\mathbf{u}'\delta_{ij}\right),\#(5)$$

where $i,j = 1,2,3$. ξ and η denote shear and bulk water kinematic viscosity coefficients. p' is the pressure fluctuations. Following the statistical mechanics theory [4], the stochastic stress tensor in small control space-time volume $(\delta V,\delta t)$ is evaluated as

$$\Pi'_{ij} = \sqrt{\frac{2k_bT}{\delta t\delta V}}\left(\sqrt{2}\sqrt{\eta} \cdot \mathrm{G}_{\mathrm{ij}}^{\mathrm{S}} + \sqrt{D}\sqrt{\xi} \cdot \mathrm{tr}[\mathbf{G}] \cdot \frac{\mathrm{E_{ij}}}{\mathrm{D}}\right)\#(6)$$

where $\mathbf{G}$ is a random Gaussian matrix whose mean is zero, $\mathrm{G}_{\mathrm{ij}}^{\mathrm{S}} = \frac{G_{ij} + G_{ij}^{T}}{2} - \mathrm{tr}[\mathbf{G}] \cdot \frac{\mathrm{E}_{\mathrm{ij}}}{\mathrm{D}}$, $\mathbf{E}$ is the unit matrix and $\mathrm{tr}[\mathbf{G}] = G_{11} + G_{22} + G_{33}$, which stands for the trace of the matrix, T and k_b are the temperature and Boltzmann constant respectively, and the isothermal equation of state, $\mathrm{p} = \mathrm{p}(\rho)$ is assumed between pressure p and density ρ.

Inside the buffer region of the FH/MD model, which separates pure molecular dynamics region from the outer continuum hydrodynamics region, modified molecular dynamics equations are solved following the two-phase flow analogy method [5-10].

A distinct novelty of the current work is extension of the above FH/MD modelling framework to include solid boundaries inside the pure MD region, where the standard MD equations are solved, $\frac{d\mathbf{x_p}}{dt} = \mathbf{u_p}$, $\frac{d\mathbf{u_p}}{dt} = \mathbf{F_p}$ ($\mathbf{x_p}$, $\mathbf{u_p}$,$\mathbf{F_p}$) ($\mathbf{x_p}$, $\mathbf{u_p}$, and $\mathbf{F_p}$ are particle coordinate, velocity, and interatomic potential force). Outside of these regions the atomistic particle equations are modified by introducing the effective continuum forces, as follows:

$$\frac{d\mathbf{x_p}}{dt} = \mathbf{u_p} + s(\tilde{\boldsymbol{u}} - \mathbf{u_p}) + \alpha(s) \cdot \frac{\sum_{\gamma=1,6}\left(\tilde{\rho} - \sum_{p=1,N_\gamma(t)}\rho_p\right)d\mathbf{n}^r}{\sum_{p=1,N(t)} m_p} \# (7)$$

$$\frac{du_{ip}}{dt} = (1-s)F_{ip}/m_{ip} +$$
$$+ \sum_{k=1,3}\sum_{\gamma=1,6}\left(\alpha(s) \cdot \sum_{p=1,N(t)}\rho_p \cdot u_{ip}\left(\frac{\sum_{\lambda=1,6}\left(\tilde{\rho} - \sum_{p=1,N(t)}\rho_p\right)dn_k^\lambda}{\sum_{p=1,N(t)} m_p}\right)\right)dn_k^\lambda / \sum_{p=1,N(t)} m_p$$
$$+ \sum_{k=1,3}\sum_{\gamma=1,6}\left(\beta(s) \cdot \frac{1}{V}\left(\sum_{\lambda=1,6}\left(\tilde{\rho} \cdot \tilde{u}_i - \sum_{p=1,N(t)}\rho_p u_{ip}\right)dn_k^\lambda\right)\right)dn_k^\gamma / \sum_{p=1,N(t)} m_p \#$$

(8)

Here $i = 1,2,3$, $s = s(x,y,z)$ is the (user-defined) multi-scale resolution function ($s = 1$ corresponds to the continuum hydrodynamics resolution and $s = 0$ corresponds to the pure molecular dynamics regime). $(\tilde{\boldsymbol{u}},\tilde{\rho}) \equiv (\rho,\mathbf{u})^{total}$ are the macroscopic velocity and density of water. $\sum_{p=1,N(t)}\rho_p$ and $\sum_{p=1,N(t)}\rho_p u_{ip}$ are the density and momenta of water atoms averaged over a control volume of the LL-FH method. Continuum density and velocity values corresponding to the particle location

are obtained by interpolation, using the fields from adjacent control volumes. It can be noted that the third term on the right-hand-side in (7) forces the control-volume-averaged particle density to the target continuum flow density. The second and the third right-hand-side terms in (8) correspond to the effect of the diffusion forcing in the density equation and the diffusion forcing of the control-volume-averaged particle momentum to the target continuum momentum.

It can be remarked that the above modified particle equations effectively use an interpolation between the all-atom and the coarse-grained/ continuum representations of the equations of motion, which is not uncommon in other multiscale methods such as AdResS [11, 12].

In the above equations, $\alpha(s) \geq 0$ and $\beta(s) \geq 0$ are model calibrations functions, which are active in the buffer zone, $0 < s < 1$ (Fig. 1 (b)). The functions are approximated by

$$\alpha \equiv \beta = \alpha_0 \cdot s(1-s)^{\gamma}, \qquad (9)$$

where $\alpha_0 = 6 - 8\ \mathrm{nm}^2/\mathrm{s}$ and γ depends on the distance between the AFM tip and the substrate. Herein $s(x,y,z)$ and γ are defined in consistence with a solution of the inverse problem to match the force on the AFM tip from the FH/MD model with the one obtained from the pure all-atom MD solution of the same tip-mica distance for the stationary tip conditions.

To close the model, function $s = s(x,y,z)$ is defined. For the AFM tip/material substrate problem, all-atom resolution is essential around all solid boundaries including the substrate wall, the AFM tip, and the surrounding water atoms, which forms a pure MD simulation region where $s = 0$. Away from this region, the resolution is smoothly relaxed to continuum hydrodynamics. Due to the smooth transition among different resolution, the model does not suffer from the multi-scale interface effect. In order to accommodate the planar substrate and the spherical tip boundaries, the following analytical function is used

$$
s(x,y,z) = \begin{cases} 0 & z \leq Z_{MD} \\ 0 & (Z_{MD} < z < Z_{FH}) \cap (r \leq R_{MD}) \\ (S_z \times S_r)/S_{max} & (Z_{MD} < z < Z_{FH}) \cap (R_{MD} < r < R_{FH}) \\ S_z & (Z_{MD} < z < Z_{FH}) \cap (r \geq R_{FH}) \\ S_{max} & z \geq Z_{FH} \end{cases} \#(10)
$$

where S_z and S_r are

$$
S_z = \frac{z - Z_{MD}}{Z_{FH} - Z_{MD}} S_{max} \#(11)
$$

$$
S_r = \frac{r - R_{MD}}{R_{FH} - R_{MD}} S_{max}. \#(12)
$$

Here $r = (x - c_x)^2 + (y - c_y)^2 + (z - c_z)^2$ corresponds to the distance from the centre of the spherical tip, which coordinates are (c_x, c_y, c_z). The maximum value is set to $S_{max} = 0.9$.

For validation in the stationary regime, the solution of the multiscale model is compared with solution of the all-atom molecular dynamics model with periodic boundary conditions. Fig. S1 (a) compares the AFM force-distance curve obtain with the suggested multiscale model with the all-atom MD solution. The error bar is included, which corresponds to the difference between the total force on the AFM tip and its mirror image in the upper part of the symmetric simulation domain. The force on the AFM tip is averaged over 2 ns period, which averaging window was found sufficient for obtaining a well-converged solution by comparison with the results of the same model averaged over 3 ns (See Fig. S1 (b)).

In consistence with the water-mica [13, 14] AFM experiment and other all-atom MD simulations [15, 16], the current MD modelling shows a strong repulsive force at distances smaller than 0.3 nm. As the distance increases to 0.4 nm, the repulsive force switches to attraction in agreement with the water-mica experiment [13]. Notably, the developed MD model of the mica-water-AFM system predicts the peak of the attraction force to be around 0.5 pN, which is in very good agreement with the experiment [13]. It can be remarked that this attraction force is not commonly reported in MD water-

mica simulations, such as in [15, 16] due to the loss of K-cations from the mica surface to the aqueous solution. The cation loss leads to a smearing of the force-distance curve, as confirmed by the experiment [17]. To prevent this effect in the current mica model, K-cations are constrained so that they vibrate around the equilibrium position but cannot leave the mica surface. The attractive dip of the AFM force curve is followed by a small repulsion peak at 0.575 nm corresponding to the honeycomb-like first hydration layer on the mica surface [14, 15]. After this peak the AFM force begins to decrease to zero, once the interfacial water effects become negligible. Notably, the comparison between the multiscale FH/MD model and the reference all-atom MD solution is excellent.

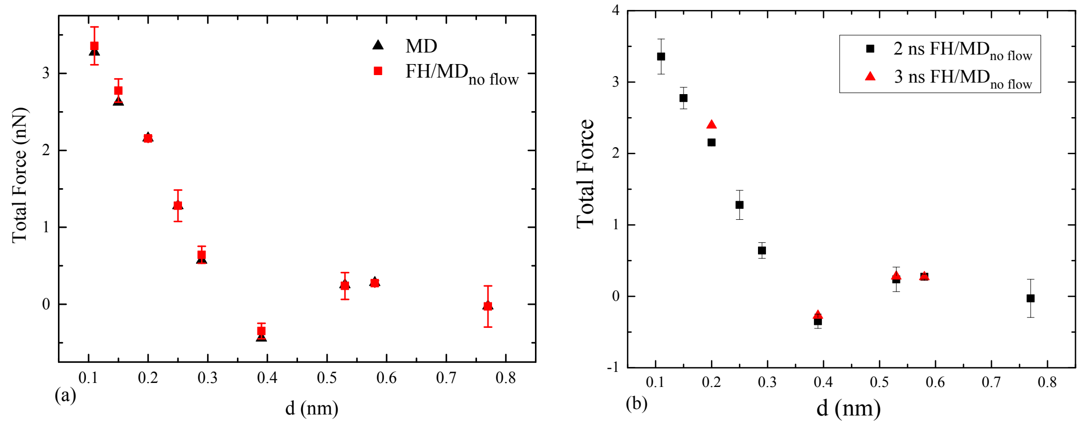

Fig. S1 Validation of force-distance curve from FH/MD model and corresponding simulation time in which the total force is obtained for stationary AFM simulation. The bars show the statistical uncertainty. (a) Comparison of force-distance curves between the MD and FH/MD solutions. The force represents the total force on the AFM tip when there is no flow. (b) Comparation of the force-distance averaged over 2 ns and over 3 ns from FH/MD model.

2. The fluctuations of the force-distance curve due to Brownian motion and the water number density above the moving AFM tip depending on the tip speed

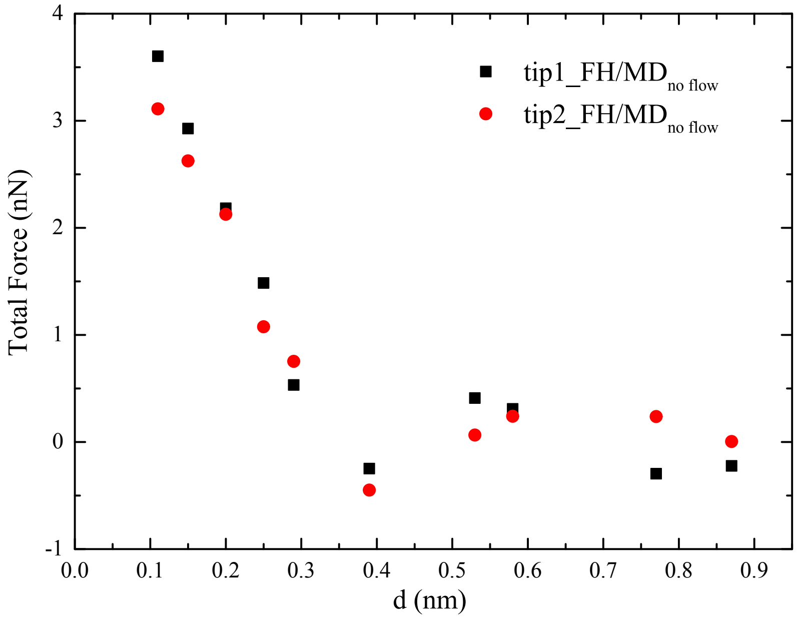

Fig. S2 Force-distance AFM curve obtained from the FH/MD model for the zero tip velocity computed for the spherical tip (tip1, solid squares) and its mirror image (tip2, slid rounds). The difference between the two sets corresponds to the statistical uncertainty due to the small size of the statistical ensemble. The absolute value of this difference is used as the error, which for different tip velocities is included in Fig. 4 (b) of the main body of the article.

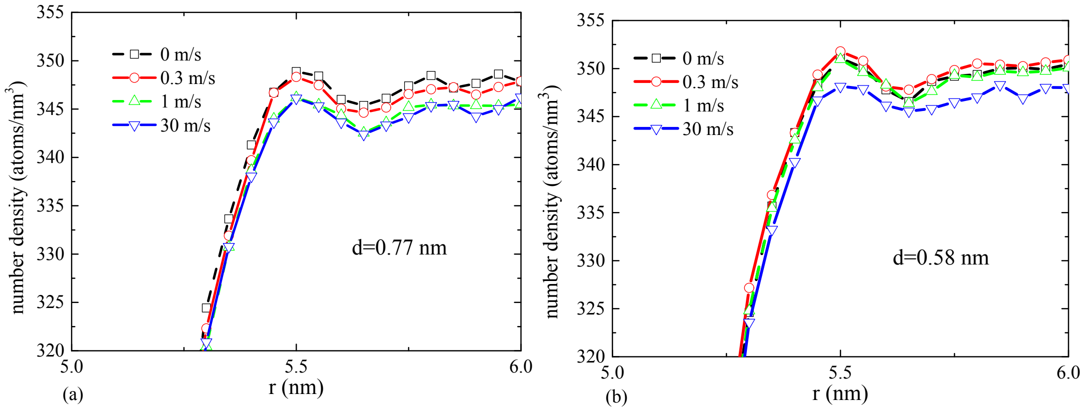

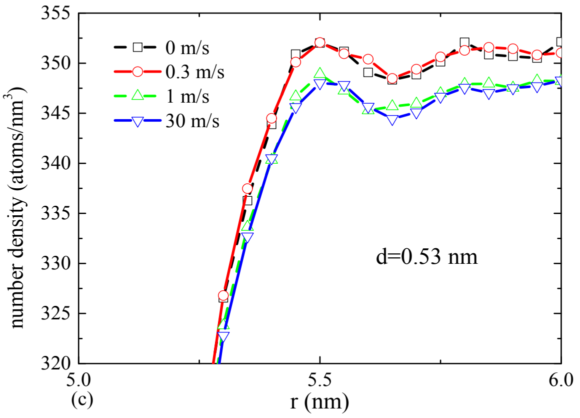

Fig. S3 The radial distribution of the number density of water atoms around the top half of the spherical AFM tip (away from the mica plane) as a function of the radial distance from the centre of the AFM tip at different mica-tip distances, *d*: (a) 0.77 nm, (b) 0.58 nm, and (c) 0.53 nm. 5 nm corresponds to the radial coordinate of the tip surface. Note the decrease of water density around the moving tip as the tip velocity increases.

3. Validation of the hybrid FH/MD method for a test molecular system with moving the atomistic/continuum interface

Following [18], the FH/MD method is applied to simulate a small peptide diffusing in water. A cubic simulation domain (8 x 8 x 8) nm3 filled with water atoms and periodic boundary conditions in all three directions is considered. In the test, the same MD water in room temperature conditions is used as for the simulation in the main paper. Instead of the AFM tip and mica substrate, for this test system, a small protein corresponding to the zwitterionic form of dialanine is introduced in the water volume. The dialanine molecule compromises two amino acid residues and is a popular system in bio-molecular research and simulated with the GROMOS 54A7 Force Field [19]. Two situations are considered: the dialanine molecule diffusion in water with and with switching on a non-uniform hydrodynamic flow. For the latter, the classical parabolic Poiseuille flow profile is considered. The analytical Poiseuille flow solution is treated in accordance with Eqs (1)-(6) and similar to the Stokes solution for the sphere/wall problem in the main FH/MD simulations of this article. The validation of the FH/MD solutions is performed by first computing the molecular diffusion coefficient and

comparing it with the value obtained from the reference all-atom MD simulation performed in the same periodic domain. Then, for the flow case, the local hydrodynamic water velocity is computed by averaging the MD particles in each control volume from the pure MD region to the hybrid zone dominated by hydrodynamics and compared with the analytical Poiseuille solution.

Fig. S4 shows the set-up of the hybrid FH/MD method where the centre of pure MD zone is fixed at the center of mass of the peptide molecule, thereby also moving with its centre of mass. The size of the pure MD zone and the hybrid FH/MD zone is selected so that they span over at least a few cut-off radii of the Wan der Waals forces, similar to how the hybrid FH/MD model was adjusted to simulate the AFM problem in the main article. Away from the zone of interest – the diffusing peptide molecule and its water shell, water atoms are driven by fluctuating hydrodynamics with or without considering the Poiseuille flow velocity profile.

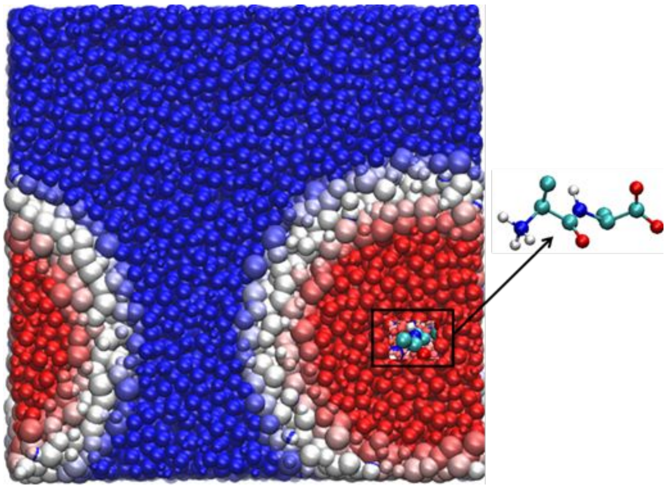

Fig. S4 The simulation domain of the hybrid multiscale method. The center of the FH/MD model is locked to the center of pure MD region and moves with the diffusing peptide. The red region is pure atomistic MD region, the white and blue region are buffer and continuum regions. Reproduced with permission from reference [18]. Copyright 2021 Elsevier.

Fig. S5a shows the mean square displacement (MSD) of the peptide molecule obtained from the hybrid FH/MD model and the reference all-atom MD simulation in

the no-flow case. The resulting molecular diffusion coefficients are computed from the slope of the MSD trajectory in accordance with the Einstein relation [20]. The FH/MD model predicts a diffusion coefficient of 0.87 x 10^{-9} m^2/s which is close to the value predicted from the reference all-atom MD simulation, 0.77 x 10^{-9} m^2/s. This demonstrates that despite the moving multi-scale interface the FH/MD can capture suitable molecular transport properties such as diffusion coefficient very well.

Fig.5b shows results of the FH/MD simulations for the Poiseuille flow case, where several maximum values of the same parabolic velocity profile were considered, U_{max} =30, 40, 55, 70, 75 and 100 m/s, which are in the order of the fastest moving AFM tip considered in the main part of the article. The flow is in the x-direction while the z-direction is along the velocity gradient. In all computed flow cases, the cell-averaged velocities of MD particles along the velocity gradient show good collapse to the analytical profile, when normalized by the peak velocity profile value. No numerical artefacts such as oscillations are notable despite multiple crossings of the velocity profile by the moving multi-scale interface of the FH/MD model.

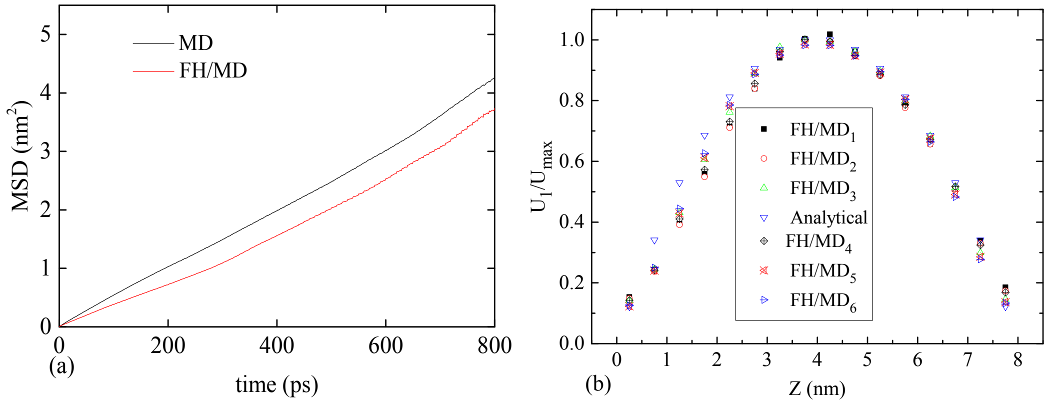

Fig. S5 Results of the no-flow diffusion test - comparison of the MSD trajectories of the peptide molecule computed with the FH/MD model and the reference all-atom MD solution (a) and the Poiseuille flow test - comparison of the mean-flow velocity profiles obtained by averaging the MD particle velocities for different maximum flow velocities, 30 m/s (FH/MD_1), 40 m/s (FH/MD_2), 55 m/s (FH/MD_3), 70 m/s (FH/MD_4), 75 m/s (FH/MD_5) and 100 m/s (FH/MD_6) (b). Reproduced with permission from reference [18]. Copyright 2021 Elsevier.

(1) Hu, X.; Nanney, W.; Umeda, K.; Ye, T.; Martini, A. Combined Experimental and Simulation Study of Amplitude Modulation Atomic Force Microscopy Measurements of Self-Assembled Monolayers in Water. Langmuir 2018, 34 (33), 9627-9633. DOI: 10.1021/acs.langmuir.8b01609.

(2) Brenner, H. The slow motion of a sphere through a viscous fluid towards a plane surface. Chem. Eng. Sci. 1961, 16 (3), 242-251. DOI: https://doi.org/10.1016/0009-2509(61)80035-3.

(3) Markesteijn, A. P.; Karabasov, S. A.; Glotov, V. Y.; Goloviznin, V. M. A new non-linear two-time-level Central Leapfrog scheme in staggered conservation–flux variables for fluctuating hydrodynamics equations with GPU implementation. Comput. Methods Appl. Mech. Eng. 2014, 281, 29-53. DOI: https://doi.org/10.1016/j.cma.2014.07.027.

(4) L. D. Landau, E. M. L. Statistical Physics; Elsevier, 1980.

(5) Korotkin, I.; Nerukh, D.; Tarasova, E.; Farafonov, V.; Karabasov, S. Two-phase flow analogy as an effective boundary condition for modelling liquids at atomistic resolution. J. Comput. Sci. 2016, 17, Part 2, 446-456. DOI: http://dx.doi.org/10.1016/j.jocs.2016.03.012.

(6) Hu, J.; Korotkin, I. A.; Karabasov, S. A. Hybrid multiscale simulation reveals focusing of a diffusing peptide molecule by parallel shear flow in water. J. Mol. Liq. 2019, 280, 285-297. DOI: https://doi.org/10.1016/j.molliq.2019.01.152.

(7) Tarasova, E.; Korotkin, I.; Farafonov, V.; Karabasov, S.; Nerukh, D. Complete virus capsid at all-atom resolution: Simulations using molecular dynamics and hybrid molecular dynamics/hydrodynamics methods reveal semipermeable membrane function. J. Mol. Liq. 2017, 245, 109-114. DOI: https://doi.org/10.1016/j.molliq.2017.06.124.

(8) Korotkin, I.; Karabasov, S.; Nerukh, D.; Markesteijn, A.; Scukins, A.; Farafonov, V.; Pavlov, E. A hybrid molecular dynamics/fluctuating hydrodynamics method for modelling liquids at multiple scales in space and time. J. Chem. Phys. 2015, 143 (1), 014110.

(9) Pavlov, E.; Taiji, M.; Scukins, A.; Markesteijn, A.; Karabasov, S.; Nerukh, D. Visualising and controlling the flow in biomolecular systems at and between multiple scales: from atoms to hydrodynamics at different locations in time and space. Faraday Discussions 2014, 169 (0), 285-302, 10.1039/C3FD00159H. DOI: 10.1039/C3FD00159H.

(10) Scukins, A.; Nerukh, D.; Pavlov, E.; Karabasov, S.; Markesteijn, A. Multiscale molecular dynamics/hydrodynamics implementation of two dimensional “Mercedes Benz” water model. Eur. Phys. J.: Spec. Top. 2015, 224 (12), 2217-2238. DOI: 10.1140/epjst/e2015-02409-8.

(11) Praprotnik, M.; Site, L. D.; Kremer, K. Adaptive resolution molecular-dynamics simulation: Changing the degrees of freedom on the fly. J. Chem. Phys. 2005, 123 (22), 224106. DOI: 10.1063/1.2132286.

(12) Praprotnik, M.; Site, L. D.; Kremer, K. Multiscale Simulation of Soft Matter: From Scale Bridging to Adaptive Resolution. Annu. Rev. Phys. Chem. 2008, 59 (1), 545-571. DOI: 10.1146/annurev.physchem.59.032607.093707.

(13) Fukuma, T.; Ueda, Y.; Yoshioka, S.; Asakawa, H. Atomic-Scale Distribution of Water Molecules at the Mica-Water Interface Visualized by Three-Dimensional Scanning Force Microscopy. Phys. Rev. Lett. 2010, 104 (1), 016101. DOI: 10.1103/PhysRevLett.104.016101.

(14) Kobayashi, K.; Oyabu, N.; Kimura, K.; Ido, S.; Suzuki, K.; Imai, T.; Tagami, K.; Tsukada, M.; Yamada, H. Visualization of hydration layers on muscovite mica in aqueous solution by frequency-modulation atomic force microscopy. J. Chem. Phys. 2013, 138 (18), 184704. DOI: 10.1063/1.4803742.

(15) Kobayashi, K.; Liang, Y.; Amano, K.-i.; Murata, S.; Matsuoka, T.; Takahashi, S.; Nishi, N.; Sakka, T. Molecular Dynamics Simulation of Atomic Force Microscopy at the Water–Muscovite Interface: Hydration Layer Structure and Force Analysis. Langmuir 2016, 32 (15), 3608-3616. DOI: 10.1021/acs.langmuir.5b04277.

(16) Tsukada, M.; Watanabe, N.; Harada, M.; Tagami, K. Theoretical simulation of noncontact atomic force microscopy in liquids. J. Vac. Sci. Technol. B 2010, 28 (3), C4C1-C4C4. DOI: 10.1116/1.3430541.

(17) Martin-Jimenez, D.; Garcia, R. Identification of Single Adsorbed Cations on Mica–Liquid Interfaces by 3D Force Microscopy. J. Phys. Chem. Lett. 2017, 8 (23), 5707-5711. DOI: 10.1021/acs.jpclett.7b02671.

(18) Li, F.; Korotkin, I.; Farafonov, V.; Karabasov, S. A. Lateral migration of peptides in transversely sheared flows in water: An atomistic-scale-resolving simulation. J. Mol. Liq. 2021, 337, 116111. DOI: https://doi.org/10.1016/j.molliq.2021.116111.

(19) Huang, W.; Lin, Z.; van Gunsteren, W. F. Validation of the GROMOS 54A7 Force Field with Respect to β-Peptide Folding. J. Chem. Theory Comput. 2011, 7 (5), 1237-1243. DOI: 10.1021/ct100747y.

(20) Einstein, A. On the Motion of Small Particles Suspended in Liquids at RestRequired by the Molecular-Kinetic Theory of Heat. Annalen der Physik 1905, 322, 549-560.
